# RETRACTED ARTICLE: BrRNE cleaves RNA in chloroplasts, regulating retrograde signals in *Brassica rapa* L. ssp. *pekinensis*

**DOI:** 10.1007/s00122-021-03905-z

**Published:** 2021-07-22

**Authors:** Xiaomeng Zhang, Xing Li, Wei Ma, Mengyang Liu, Shu Zhang, Yan Li, Yin Lu, Daling Feng, Shuxing Shen, Jianjun Zhao

**Affiliations:** State Key Laboratory of North China Crop Improvement and Regulation, Key Laboratory of Vegetable Germplasm Innovation and Utilization of Hebei, Collaborative Innovation Center of Vegetable Industry in Hebei, College of Horticulture, Hebei Agricultural University, Baoding, 071000 China

The Editor-in-Chief has retracted this article because there are anomalies in the Northern blots shown in Figure 3C. The data reported in this article are therefore unreliable. The authors have been invited to submit a new manuscript for peer review. All authors agree to this retraction. The online version of this article contains the full text of the retracted article as Supplementary Information.

## Supplementary Information

Below is the link to the electronic supplementary material.

Former article version (PDF 5363 kb)

