# Supplementary material for: RETRACTED ARTICLE: BrRNE cleaves RNA in chloroplasts, regulating retrograde signals in Brassica rapa L. ssp. pekinensis
Source: Theor Appl Genet. 2021 Jul 22;135(5):1811. doi: 10.1007/s00122-021-03905-z (PMC9110522; doi:10.1007/s00122-021-03905-z)
Supplement: Supplementary file 1 — Former article version (PDF 5363 kb) [file 122_2021_3905_MOESM1_ESM.pdf]

Theoretical and Applied Genetics
https://doi.org/10.1007/s00122-021-03905-z

ORIGINAL ARTICLE

RETRACTED ARTICLE

BrRNE cleaves RNA in chloroplasts, regulating retrograde signals in *Brassica rapa* L. ssp. *pekinensis*

Xiaomeng Zhang[1] · Xing Li[1] · Wei Ma[1] · Mengyang Liu[1] · Shu Zhang[1] · Yan Li[1] · Yin Lu[1] · Daling Feng[1] · Shuxing Shen[1] · Jianjun Zhao[1]

Received: 23 May 2021 / Accepted: 30 June 2021
© The Author(s) 2021

Abstract

Key message **Brassica rapa RNE participates in the processing of polycistron[illegible] precursor transcripts into mature monocistronic mRNAs in plastids, thereby sending strong retrograde signals.**

Abstract Leaf color is one of the most important agronomic traits for Chines[illegible] cabbage. Not only is it closely linked to photosynthesis, thereby affecting plant growth, but it also influences consum[illegible]ence in the marketplace. A pale-green mutant *rne* was produced by EMS mutagenesis of Chinese cabbage inbred line A[illegible] Chlorophyll content, photosynthetic rate, actual quantum efficiency (φPSII), and maximum quantum efficiency [illegible] of photosystem II (PSII) were all reduced in *rne* plants. Genetic analysis indicated that the pale-green trait was controlled by a pair of recessive alleles. Using mixed pool sequencing of F_2 individuals derived from an *rne* × wild-type cross, we identified the essential gene *Brassica rapa RNase E* (*BrRNE*), which is responsible for chloroplast developm[illegible]RNE cleaves polycistronic RNA in Chinese cabbage A03 plastids, but *rne* plants are defective in RNA processin[illegible]nd sh[illegible]w reduced translation levels of the seven plastid genes, *BrpsaB*, *BrpsaA*, *BrpsbA*, *BrpsbD*, *BrpsbB*, *BrpetA*, [illegible] *Bry*[illegible] Abnormal RNA processing in the plastids sends retrograde signals that markedly regulate the expression of [illegible]cle[illegible]r genes, upregulating genes that participate in ribosome and DNA replication pathways and repressing photosynth[illegible]sis-a[illegible]ciated nuclear genes (*PhANG*s). Our study reveals a new regulatory mechanism by which plastid RNA cleavage [illegible]fluences [illegible]lastid development and leaf color, sending retrograde signals that affect the expression of nuclear genes in *Brassic*[illegible]

Introduction

Photosynthesis is essential for plant growth and development, and chlorophyll (Chl) is the main pigment that absorbs light energy and drives electron transport in the photosynthetic reaction centers of higher plants (Tanaka and Tanaka 2006). Defects in Chl biosynthesis, degradation, or other related pathways often result in leaf color mutants. These mutants are widely distributed in nature and produce a variety of phenotypes, including albino, virescent, chlorina, xanthas, maculate, striped, and dark green (Jung et al. 2003; Manjaya 2009; Singh and Ikehashi 1981). Leaf color mutants are ideal genetic materials with which to study the molecular mechanisms of plant photosynthesis and chloroplast development.

Leaf color mutants are induced by multiple genetic and environmental factors, among which genetic change plays a decisive role. Previous studies have reported that the main molecular mechanisms of chl-related mutations are: (1) mutations in genes of the chl biosynthesis pathway

Communicated by Li[illegible]Jiang.

Xiaomeng Zhang, X[illegible]i, Wei Ma These authors contributed equally to this work.

✉ Wei M[illegible]
mawei07[illegible]16[illegible].com

✉ Shu[illegible]ng Shen
[illegible]nsx@[illegible]ebau.edu.cn

✉ Jian[illegible]n Zhao
jjz1971@aliyun.com

1 State Key Laboratory of North China Crop Improvement and Regulation, Key Laboratory of Vegetable Germplasm Innovation and Utilization of Hebei, Collaborative Innovation Center of Vegetable Industry in Hebei, College of Horticulture, Hebei Agricultural University, Baoding 071000, China

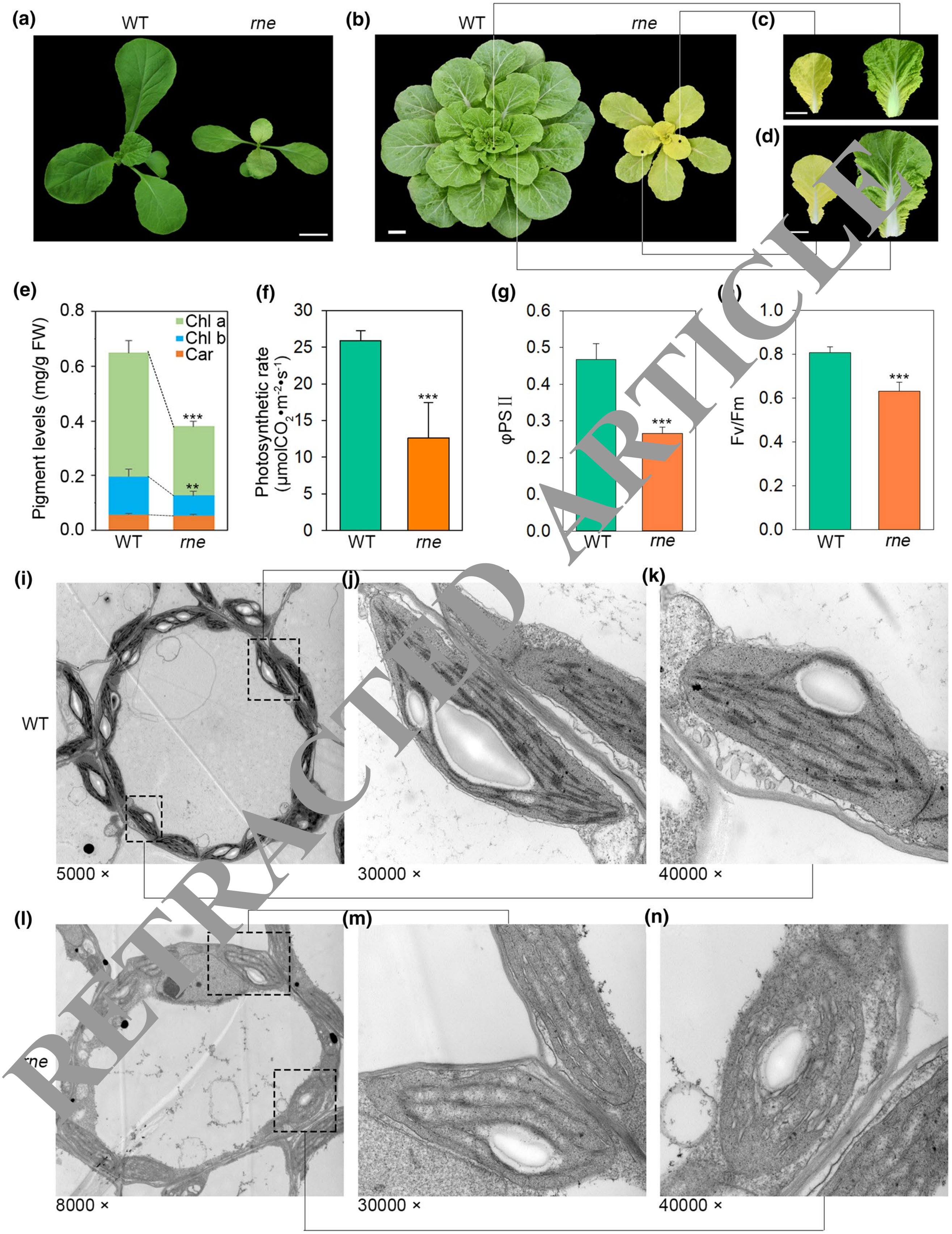
(a)
WT
rne
(b)
WT
rne
(c)
(d)
(e)
Pigment levels (mg/g FW)
Chl a
Chl b
Car
***
**
WT
rne
(f)
Photosynthetic rate (μmolCO2•m-2•s-1)
***
WT
rne
(g)
φPSII
***
WT
rne
Fv/Fm
***
WT
rne
(i)
WT
5000 ×
(j)
30000 ×
(k)
40000 ×
(l)
8000 ×
(m)
30000 ×
(n)
40000 ×
RETRACTED ARTICLE

RETRACTED ARTICLE

◂**Fig. 1** Phenotypic characterization and morphology of the pale-green *rne* mutant. **a–d** The phenotype of wild-type and *rne* plants at the seedling stage (**a**) and the rosette stage (**b–d**). Bars = 2 cm. **e–h** Chlorophylls and carotenoids contents (**e**), photosynthetic rate (**f**), actual quantum efficiency (φPSII) (**g**), and maximum quantum efficiency (Fv/Fm) (**h**) of wild-type and *rne* leaves at the rosette stage. Chl a, chlorophyll a; Chl b, chlorophyll b; Car, carotenoids. Data represent mean ± s.d. of three independent biological replicates (from different seedling leaves). ***P*-value < 0.01 (Student's t-test). ****P*-value < 0.001 (Student's t-test). **i–n** transmission electron microscopy images of cells from wild-type (**i–k**) and *rne* (**l–n**) leaves at the rosette stage. Magnification is indicated on the bottom of each image

such as *CAO* (Tanaka 1998), *CHLH* (Jung et al. 2003), *DVR* (Nagata et al. 2005), *PORB*, and *PORC* (Frick et al. 2003; Beale 2005); (2) mutations in heme metabolism genes that influence chromophore biosynthesis (Parks and Quail 1991; Terry and Kendrick 1999; Muramoto et al. 1999); and (3) mutations in genes related to chloroplast differentiation and development, including *ALBINO3* (Sundberg et al. 1997), *PGP1* (Babiychuk et al. 2003), and *RNE* (Mudd et al. 2008; Walter et al. 2010; Schein et al. 2008). Significant progress has been made in the study of leaf color in model plants such as *Arabidopsis thaliana*, rice, and maize, but there have been fewer such studies on vegetable crops. The development of next-generation sequencing technology, combined with classical genetic methods, has enabled the discovery of leaf color-related genes in vegetables. For example, six genes (*LG1_162414*, *GST*, *CAD*, *MYB113*, *bHLH*[illegible], and *ANS*) have been found to regulate the diversity of leaf [illegible] in lettuce (Zhang et al. 2017a, b).

Chinese cabbage (*Brassica rapa* L.ssp. *pekinensis*) originated in central China; it is an important vegetable crop that is most widely grown in Asia. [illegible] *rapa* is among the most closely related species to the [illegible] plant *Arabidopsis thaliana*, in which research [illegible] leaf color development has laid a foundation for its study in *Brassicas*. However, the Chinese cabbage genome has undergone triplication, and some genes are [illegible] multiple copies, making the regulation of leaf color development more complex. To date, a variety of [illegible] color mutants have been obtained in *Brassica* crops such [illegible] cauliflower (Chiu et al. 2010), oilseed rape (Zhao et al. 2001), pakchoi (Wang et al. 2018), and Chinese cabbage (Wang et al. 2014). The orange inner leaf [illegible] Chinese cabbage is controlled by a single recessive gene ([illegible]) that causes abnormal accumulation of carotene (Feng et al. 2012). *BrCRTISO*, a carotenoid isomerase specifically required for carotenoid biosynthesis, was identified as a candidate gene for the control of orange inner leaf (Zhang et al. 2013, 2015). The purple-leaf mutation of Chinese cabbage is regulated by a pair of dominant alleles, *BrPur* (Wang et al. 2014) and *BrMYB2* (He et al. 2020). In non-heading Chinese cabbage, the evergreen leaf mutant phenotype is controlled by a pair of recessive nuclear alleles, *Brnye1* (Wang et al. 2018) and *BrNYM1* (Wang et al. 2020). Overall, despite the identification of some leaf color-related genes in Chinese cabbage, little is known about their regulatory mechanisms.

Here, we identified a pale-green Chinese cabbage mutant in an EMS-mutagenized population. It retained a pale-green leaf color and exhibited reduced chlorophyll content relative to the wild-type during the seedling stage. We discovered that the mutation responsible for the pale-green trait was a stop-gain base pair change [illegible] chromosome A07. Compared with the wild-type, the mutant had an RNA processing defect in the chloroplast and exhibited repressed plastid gene translation. These results revealed that the function of *BrRNE* [illegible] to regulate plastid RNA cleavage and translation. Our findings may aid in the development of new tools for the genetic improvement of horticultural crops.

Materials and methods

Plant materials

[illegible] mutant library of Chinese cabbage was generated by treating the seeds of inbred line A03 with ethyl methanesulfonate (EMS) (Lu et al. 2016), and the pale-green mutants (*rne* and *rne2*) were isolated from the M_6 generation. A03 × *rne* F_1 and F_2 populations were developed and used for the genetic analysis of mutant traits. The plants were grown in green house at Hebei Agricultural University in Baoding (115.47 E, 38.87 N), China, in 2016 and 2018. In August 2016, 60 M_6 plants of A03 and *rne* were grown in the same green house at Hebei Agricultural University. At the rosette stage (40 days after sowing), the soft portion of the second leaf from the interior, excluding the leaf petiole, was sampled (Fig. 1c). All leaf samples were snap frozen in liquid nitrogen and stored at − 80 °C for RNA extraction or immunoblotting.

Chlorophyll and carotenoid contents, photosynthetic rate, φPSII and Fv/Fm

At the rosette stage, chlorophyll and carotenoids contents of rosette leaves were measured according to Lichtenthaler et al. (1983). Photosynthetic rate of rosette leaves was determined using the LI-COR LI-6400XT portable photosynthesis system (LI-COR Biosciences, Lincoln, NE, USA). φPSII and Fv/Fm were measured using the PAM-2500 portable chlorophyll fluorescence apparatus (Walz, Germany). All

measurements were conducted with three independent biological replicates (from different seedling leaves).

Transmission Electron Microscopy (TEM)

For TEM, the middle portion of the rosette leaves at the rosette stage was cut into 1-mm^2 fragments and fixed in 2.5% glutaraldehyde in 0.1 M sodium phosphate buffer (PBS, pH 7.3) for at least 4 h at 4 °C. The tissue was post-fixed with 1% osmium tetroxide for 2 h after extensive washing in PBS at room temperature. After dehydration in a graded ethanol series, the tissue was infiltrated using a Spurr Low Viscosity Embedding Kit (Sigma-Aldrich). Ultrathin sections (70–90 nm) were cut with a diamond knife on a Leica EM UC7 Ultramicrotome (Leica Microsystems) and examined using a Hitachi H7600 transmission electron microscope at 75–100 kV.

Phylogenetic analysis

RNE protein sequences from flowering plants were obtained from JGI Phytozome v12 (https://phytozome.jgi.doe.gov/pz/portal.html). Amino acid sequences were aligned using Clustal X with default settings for multiple protein alignment. A maximum likelihood phylogenetic tree of RNE proteins from *Arabidopsis thaliana*, *B. rapa*, *Malus domestica*, *Glycine max*, *Manihot esculenta*, *Populus trichocarpa*, *Kalanchoe fedtschenkoi*, *Sorghum bicolor*, *Setaria italica*, *Oryza [illegible]*, *Zostera marina*, and *Amborella trichopoda* was constr[illegible]ed using PhyML (Guindon et al. 2010) with S[illegible]-like branch supports.

RNA extraction and qPCR an[illegible]lysis

Total RNA was extracted using [illegible] reagent (Invitrogen). RNA was reverse transcribed into cDNA using the PrimeScript RT Reagent Kit with [illegible]DNA Eraser (Takara) and used for qRT–PCR. All q[illegible] experiments were performed in three biological replica[illegible] and three technical replicates using SYBR Gree[illegible] Master Mix (Vazyme) and the CFX Connect Real-Time PCR [illegible]stem (BioRad). The procedure for qRT–PCR was as follows: 10 min at 95 ℃, followed by 40 cycles of [illegible] at 95 ℃, 10 s at 57 ℃, and 10 s at 72 ℃. Then, a mel[illegible] curve [illegible]s generated by gradually increasing the temperature [illegible] 95 ℃ to ensure the specificity of PCR amplification. [illegible]lative gene expression levels were calculated with the $2^{-\Delta CT}$ method. Specific primer sequences are summarized in Table S2.

Subcellular localization in *Nicotiana benthamiana*

The coding sequences of *BrRNE* and *BrmRNE* were amplified by PCR from the cDNA using the KOD-Plus-Neo enzyme (TOYOBO). Purified PCR products were cloned separately into the N-terminus of eGFP between the NcoI and SpeI restriction sites under the cont[illegible] of the CaMV 35S promoter using the In-Fusion HD Cloning [illegible]it (Takara), and Sanger-sequencing was used t[illegible] validate the resulting constructs. The validated expressio[illegible] vectors were transformed into the *Agrobacteriu[illegible] tumefacie[illegible]* strain GV3101. Five-week-old fully expande[illegible] *N. benthamiana* leaves were infiltrated with 35S::*Br[illegible]*-G[illegible] 35S::*BrmRNE*-GFP, and 35S::GFP (an empty [illegible]ontrol) that had been diluted to an OD_{600} of 0.6[illegible]8 with a [illegible]olution of 10 mM MES (pH 5.6), 150 μM ace[illegible]one, and 10 mM $MgCl_2$. Two days after infiltrat[illegible] small le[illegible]f sections were examined under a Leica T[illegible] SP[illegible] confocal microscope. A 488 nm argon laser and a P[illegible] detector with emission bandwidth set to 500–5[illegible] nm were used to monitor GFP fluorescence. Specific pr[illegible] [illegible]quences are presented in Table S3.

[illegible]estern blotting

[illegible]ntact chloroplasts of 4-week-old wild-type and *rne* seedlings were isolated using a Chloroplast Isolation Kit (Sigma). Chloroplasts were immediately ground in liquid nitrogen and thawed in equal volumes of extraction buffer (50 mM Tris–HCl [pH 7.5], 0.5 (v/v) β-mercaptoethanol, 150 mM NaCl, 0.1% Triton X-100, 1 mM EDTA, 1 mM 4-(2-aminoethyl)-benzenesulfonyl fluoride, 2 μg/mL aprotinin, 2 μg/mL leupeptin, and 2 μg/mL antipain) on ice for 10 min. The mixture was centrifuged at 4 °C and 15,300 g for 30 min. Supernatants were separated in equal protein concentrations and resolved by SDS-PAGE electrophoresis, then transferred to polyvinylidene difluoride membranes. Western blotting was performed using antibodies against PsbA (Agrisera AS05084), PsaA (PhytoAB PHY0368A), PsaB (PhytoAB PHY0054A), PsbD (PhytoAB PHY0060), PsbB (PhytoAB PHY0058A), PetA (PhytoAB PHY0488A), and Ycf4 (PhytoAB PHY0441S) and imaged with the Odyssey Infrared Imaging System (LI-COR). RbcL was stained by Ponceau S as internal control. Protein levels were normalized to 690 μg.

RETRACTED ARTICLE

RETRACTED ARTICLE

Northern blotting

Total RNA was extracted from wild-type and *rne* plants using TRIzol reagent (Invitrogen). For northern blotting, RNA samples (20 µg total RNA) were electrophoresed in formaldehyde-containing 1% agarose gels and blotted onto nylon membranes (Millipore). Hybridization probes were generated by direct polynucleotide synthesis and labeled with digoxin at their 5′ ends. Hybridizations were performed at 42℃ in a hybridization buffer (Roche). After anti-digoxigenin AP-conjugate (Roche) incubation, CSPD chemiluminescent substrate (Roche) was added, and images were obtained with a Tanon 5200 imaging system.

Transcriptome sequencing and data analysis

Total RNA was extracted from wild-type and *rne* plants using TRIzol reagent (Invitrogen). Transcriptome sequencing was performed as previously described (Zhao et al. 2015). *P*-values were adjusted using the Benjamini and Hochberg (1995) method, and genes with an adjusted *P*-value < 0.05 and $|\log_2(\text{FoldChange})| > 1$ were considered to be differentially expressed. GO enrichment analysis of the differentially expressed genes was performed using the GOseq R package (Young et al. 2010). GO terms with a corrected *P*-value < 0.05 were considered to be significantly enriched. The KOBAS software was used to test the statistical enrichment of differentially expressed genes in KEGG pathways (Mao et al. 2005).

MutMap and kompetitive allele-specific PCR (KASP)

MutMap genome sequencing was performed as previously described (Abe et al. 201[illegible]). The primers used for PCR sequencing of SNPs in genes are listed in Table S1. In brief, a pool containing equal amounts of DNA from 30 pale-green *rne* × wild-type F_2 plants was re-sequenced (30 × coverage). Delta SNP index = 1 was used to filter SNP loci in the mutant genes. SNP markers were used for KASP genotyping. The co-segregating SNP marker was selected by analysis of both the allelic site and the leaf color trait.

Results

Phenotypic characterization and morphology of the pale-green *rne* mutant

Pale-green *rne* plants were clearly smaller than wild-type plants and displayed a chlorotic phenotype (Fig. 1a–d). Visible chlorosis appeared initially in the cotyledons of the mutant plants (Figure S1). As the plants grew, all the true leaves became chlorotic as they emerged. When grown under photoautotrophic conditions in the soil, *rne* plants grew somewhat slowly (Fig. 1a–d) but survived well, flowering and producing seeds, albeit in limited quantities.

We next characterized the physiological basis of the *rne* mutation in detail. Chlorophyll and carotenoid contents, photosynthetic rate, and the efficiency of photosynthetic electron transport were determined by spectroscopy, infrared gas analysis, and chlorophyll fluorescence measurements. As suggested by their pale-green phenotype, *rne* plants had a reduced chlorophyll content (Fig. 1e). However, the ratio of chlorophyll a to chlorophyll b and the carotenoid content did not differ between *rne* and wild-type plants (Fig. 1e). Photosynthetic rate was also significantly reduced in *rne* plants (Fig. 1f), as were the actual quantum efficiency (φPSII) and the maximum quantum efficiency of photosystem II (PSII; Fv/Fm), which are standard measures of PSII integrity (Fig. 1g–h). These results suggested that a proportion of the PSII reaction centers in the *rne* mutant may have been damaged.

To confirm the defect in chloroplast development indicated by the pale-green leaves of *rne* plants, the true leaves of wild-type and *rne* plants were observed by transmission electron microscopy (TEM). TEM analysis revealed that chloroplasts in the leaves of wild-type seedlings contained a well-formed thylakoid system of stromal and granal thylakoids, but thylakoid formation was severely altered in the mutant. In the chloroplasts of *rne* mutant leaves, both the stromal and granal thylakoids took the form of unusual vesicle-like structures. The plastids themselves were smaller and had fewer thylakoids and shorter granal stacks than

Table 1 The segregation ratios of F_1 and F_2 individuals from an *rne* × wild-type cross in 2016 and 2018

Generation (year)	Total plants	Wild-type plants (green)	Mutant plants (pale-green)	Segregation Ratio	χ^2 value
F_1 (2016)	10	10	0	10:0	
F_1 (2018)	9	9	0	9:0	
F_2 (2016)	205	156	49	3.2:1	0.132
F_2 (2018)	265	197	68	2.9:1	0.062

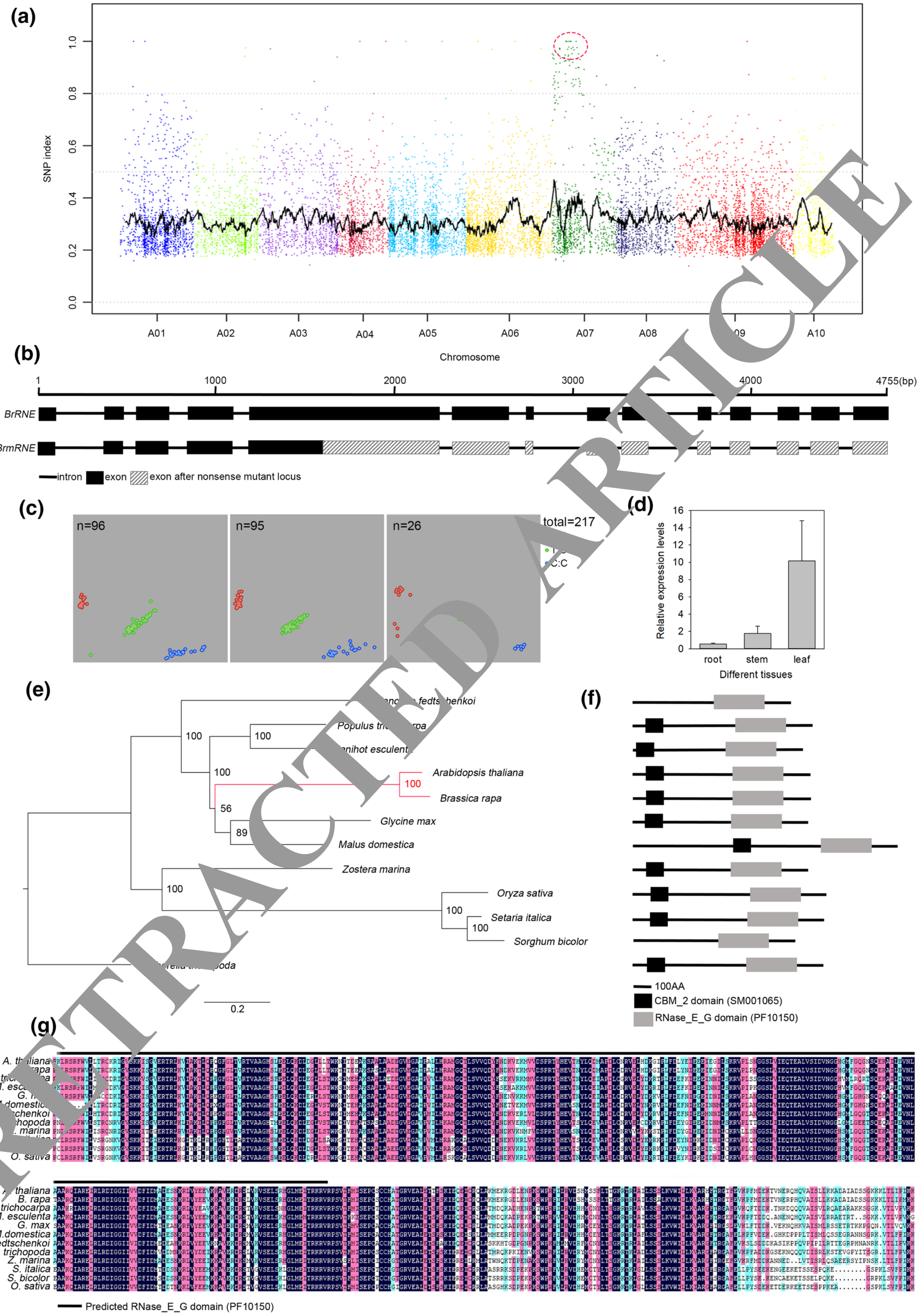

RETRACTED ARTICLE

◂**Fig. 2** Identification of *BrRNE* as responsible for the pale-green trait and characterization of RNE proteins. **a** SNP index of all SNPs on ten chromosomes in MutMap. Red circle represents the candidate SNP loci on the chromosome A07. **b** Structural diagram of the genes *BrRNE* and *BrmRNE*. **c** KASP analysis of an F_2 population that consisted of 217 plants (*rne* × wild-type) using the SNP marker in *BrRNE*. Red, allele T:T. Green, allele T:C. Blue, allele C:C. **d** Relative expression levels of *BrRNE* in the roots, stems, and leaves. The expression level of *BrACTIN7* was used as an internal control. **e** Phylogenetic tree of RNEs from 12 flowering plants. Red branches show that BrRNE exhibited the highest similarity to *Arabidopsis thaliana* RNE (AtRNE). **f** Domain organization of RNE proteins from 12 flowering plants in (**e**). The CBM_2 and RNase_E_G domains are shown in black and gray boxes, respectively. AA, Amino acid. **g** Alignment of RNE proteins from 12 flowering plants. The conserved RNase_E_G domains is marked by an upper black bold line. Bases with a black background are 100% homologous, those with a red background are 75–100% homologous, and those with a blue background are 50–75% homologous

wild-type chloroplasts (Fig. 1i–n). Nonetheless, the chloroplasts of both *rne* and wild-type plants accumulated starch granules normally (Fig. 1j–k, m–n), suggesting that the photosynthetic capacity of the *rne* plants was not strongly affected.

Inheritance of the mutant trait

Genetic analysis showed that the phenotypic traits of all F_1 plants were similar to those of the wild-type. Analysis of the F_2 populations in 2016 and 2018 showed that the proportions of wild-type and mutant plants were 3.2:1 and 2.9:1, respectively, conforming to a 3:1 ratio (χ^2 = 0.132 and 0.062, respectively) (Table 1). Therefore, we initially speculated that the pale-green trait was controlled by a pair of recessive alleles.

Identification of candidate mutant genes

DNA samples were collected from 30 recessive mutant F_2 progeny (*rne* DNA pool) and 30 wild-type plants (wild-type DNA pool) and bulked sequencing with substantial genomic coverage (30×), respectively. Comparison of sequence reads from the wild-type and *rne* DNA pools, identified a single unique genomic region that harbored a cluster of SNPs with SNP index of > 0.8 on chromosome A07 that contained 72 SNPs (Fig. 2a). Among the SNPs with SNP index of 1, only four were predicted to cause amino acid changes, including one non-synonymous and three stop-gain substitutions, representing four different mutant genes.

To verify whether the mutant phenotype was caused by a mutation in the candidate gene, sequencing primers were designed near the mutation site and used to amplify and sequence the PCR products. The results indicated that only two of the stop-gain substitutions were real (Figure S3).

Kompetitive Allele-Specific PCR (KASP) analysis was further performed in 217 F_2 progeny (*rne* × wild-type). Of the two validated SNP markers (causing stop-gain substitutions), only the SNP marker in BraA07000683 co-segregated with leaf color (Fig. 2c); 55 pale-green F_2 progeny had T:T, and 162 green F_2 progeny had T:C or C:C (108 T:C and 54 C:C). The proportion of green and pale-green plants conformed to a [illegible]:1 ratio (χ^2 = 0.014), and the proportion of T:T, T:C and C:C conformed to a 1:2:1 ratio (χ^2 = 0.021). In addition, transcript levels of BraA07000683 in roots, stems, and leaves indicated that the gene was preferentially expressed in leaves (Fig. 2d). Thus, the gene BraA07[illegible]0683 was considered to be responsible for the leaf color development phenotype.

BraA070006[illegible]3 is the homolog of the Arabidopsis RNase E/G gene *AtRNE* (AT2G04270) and was therefore named *BrRNE*. *B. rapa* contains a single RNase E/G gene in its haploid genome. The stop-gain substitution occurred before the RNase_E_G domain (PF10150) of BrRNE, causing a defect in this domain (Fig. 2b, Figure S4).

To confirm that *BrRNE* was the key gene responsible for the pale-green phenotype, we investigated the DNA sequencing data from the *Brassica rapa* EMS mutant population (Lu et al. 2016). One SNP was observed in the first base of the 11th intron of *BrRNE* in a pale-green leaf EMS line (*rne2*) (Figure S5a–c). The transcript of *BrRNE* was analyzed further, and the splice donor variant (*BrmRNE'*) of *BrRNE* was also obvious in *rne2* (Figure S5d), strongly suggesting that *BrRNE* plays an essential role in the regulation of leaf color morphology in Chinese cabbage.

To investigate putative RNE homologs in higher plants, we searched for RNE protein homologs in 12 angiosperm genomes: eight eudicot species, four monocot species, and a basal angiosperm. Domain analysis revealed that all the angiosperm RNEs contained an RNase_E_G domain, which was highly conserved in flowering plants (Fig. 2e–g). Phylogenetic analysis showed that BrRNE clustered together with Arabidopsis RNE (AtRNE) (Fig. 2e), suggesting that the functions of BrRNE in Chinese cabbage may be similar to those of AtRNE.

BrRNE cleaves RNA and affects plastid gene expression in the chloroplast

To determine the subcellular localization of the wild-type and mutant BrRNE proteins, we constructed a gene fusion of *BrRNE* or mutant *BrRNE* (*BrmRNE*) with the *GFP* gene and transformed the chimeric gene into tobacco epidermal cells. The green fluorescence of the reporter protein overlapped with the red fluorescence of chlorophyll, strongly

RETRACTED ARTICLE

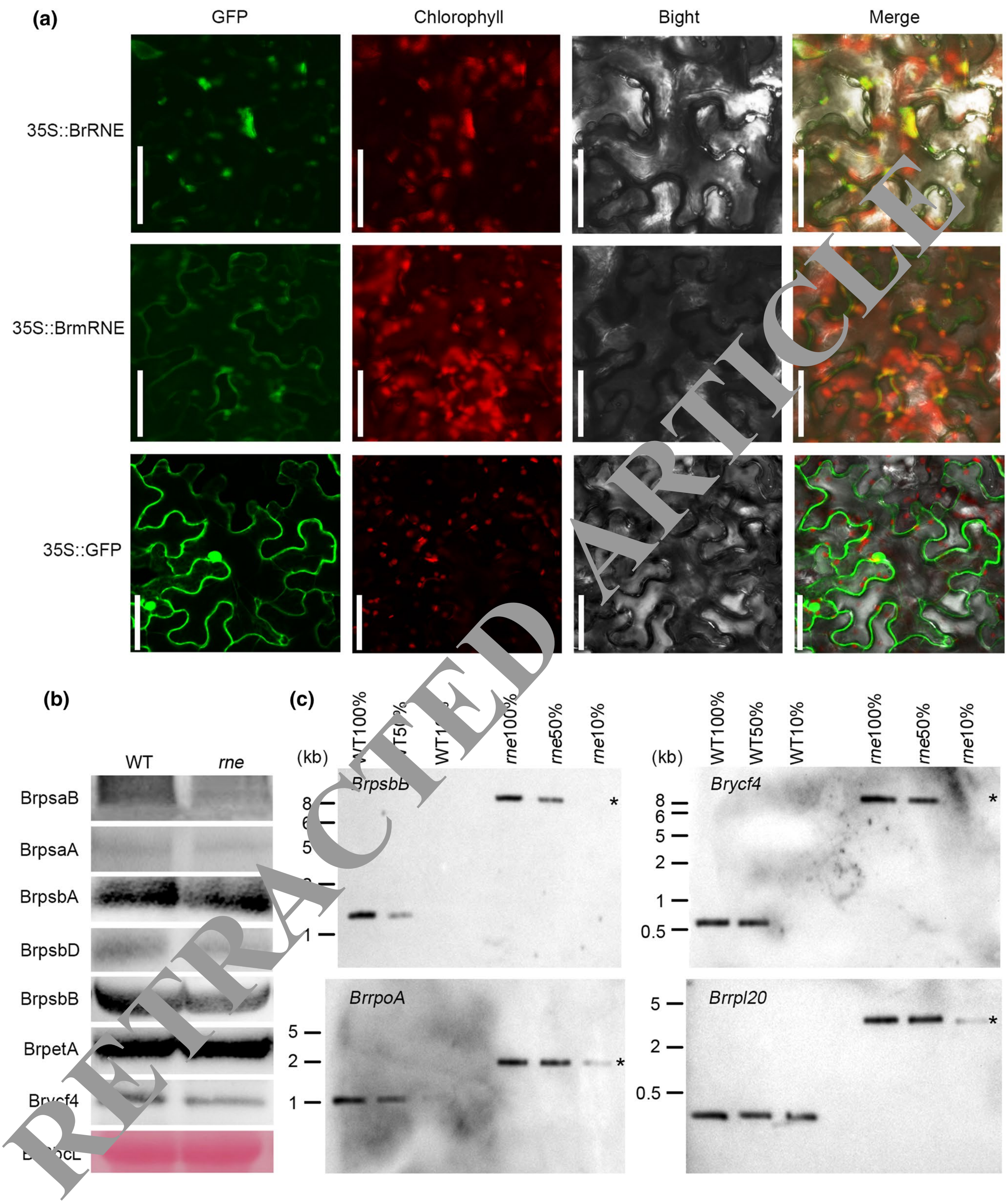

RETRACTED ARTICLE

suggesting that BrRNE was localized to the chloroplast. By contrast, BrmRNE was localized to both the chloroplast and the plasma membrane, and free GFP signal was observed throughout the entire cell (Fig. 3a). This result is consistent with previous reports of AtRNE subcellular localization in the chloroplast (Schein et al. 2008; Mudd et al. 2008; Walter et al. 2010). Taken together, our data indicate that BrRNE is localized in plastids, suggesting that its role may be similar to that of AtRNE in plastid development.

RETRACTED ARTICLE

◂**Fig. 3** *B. rapa* RNase E (BrRNE) cleaves polycistronic RNA in Chinese cabbage plastids. **a** Subcellular localization of the BrRNE-GFP and *B. rapa* mutant RNase E (BrmRNE)-GFP fusion proteins in *Nicotiana benthamiana* epidermal cells were observed and imaged by confocal microscopy. The GFP fluorescence (GFP), the chlorophyll autofluorescence (Chlorophyll), the bright-field images, and the merged images are shown. The bottom panel shows a negative control using an empty vector with free GFP. Bars = 50 μm. **b** Accumulation of BrpsaB (a core protein of the PSI multisubunit complex comprised of BrpsaA and BrpsaB), BrpsaA (another core protein of the PSI complex), BrpsbA (the D1 protein of PSII), BrpsbD (the D2 protein of PSII), BrpsbB (the CP47 subunit of PSII), BrpetA (a cytochrome f apoprotein of PSI), and Brycf4 (a photosystem I assembly protein) determined by immunoblot analysis with specific antibodies. Rubisco stained with Ponceau S is used as a control for total chloroplast proteins. **c** RNA accumulation and processing patterns of the four plastid genes, *BrpsbB, Brycf4, BrrpoA* and *Brrpl20* in Chinese cabbage *rne* mutants. Northern blots were hybridized to specific probes for the four plastid genes. Sizes of marker bands are given in kb. Precursor transcripts that overaccumulated in *rne* plants are indicated by asterisks. For quantitative comparison, a dilution series of the wild-type (WT) and *rne* RNA samples was loaded

Endoribonuclease E (RNE) is a member of the RNase E/G ribonuclease family (Mudd et al. 2008). To determine the role of BrRNE in *B. rapa* chloroplasts, the complete *B. rapa* plastid genome sequence was downloaded from NCBI (GenBank: MT726210.1) and assembled into a circle graph (Figure S6) with the OGDRAW tool (http://ogdraw.mpimp-golm.mpg.de/, Greiner et al. 2019). The *B. rapa* plastid genome is 153,494 bp in length, contains 132 genes, and includes two large inverted repeats (IRs, IRA, and IRB) separated by large and small single-copy regions (the [illegible] and SSC) (Figure S6).

The putative defects in chloroplast RNA processing in *rne* plants were further investigated by northern blotting in wild-type and *rne* plants. We analyzed the transcription accumulation of four plastid genes, including *BrpsbB* (a PSII gene that is part of a large polycistronic transcription unit), *Brycf4* (a PSI gene that is also part of a large polycistronic transcription unit), *BrrpoA* (an RNA polymerase gene), and [illegible] (a chloroplast ribosomal gene). Apparent massive overaccumulation of unprocessed polycistronic precursor transcripts was observed in *rne* plants, suggesting that RNE is involved in processing polycistronic precursor transcripts into mature monocistronic mRNAs in *B. rapa* chloroplasts (Fig. 3c). In addition, immunoblot analyses using antibodies against BrpsaB (a core protein of PSI multisubunit complex comprised of psaA and psaB), BrpsaA (another core protein of the PSI), BrpsbA (the D1 protein of PSII), BrpsbD (the D2 protein of PSII), BrpsbB (the CP47 subunit of photosystem II), BrpetA (a cytochrome f apoprotein of photosystem I), and Brycf4 (a photosystem I assembly protein) directly confirmed their reduced accumulation (Fig. 3b), suggesting that impaired precursor processing reduced protein translation in plastids.

Abnormal plastid RNA processing causes substantial retrograde signaling to the nucleus

Changes in chloroplast development and/or gene expression can cause massive changes in nuclear gene expression (Chi et al. 2015). We further analyzed our RNA-seq data to investigate global changes in the nuclear gene expression of *rne* plants. The expression of [illegible]26 nuclear genes was increased and that of 4277 nuclear genes was reduced in *rne* plants compared with the wild-type ($P < 0.05$; $|\log_2(\text{FoldChange})| > 1$) (Figure S7).

We further categorized the differentially expressed genes using gene ontology [illegible] enrichment analysis. Genes upregulated in *rne* versus wild-type plants were significantly enriched in DNA replication (biological process), ribosome (cellular component), and structural constituent of ribosome (molecular function) (Fig. 4a). The genes upregulated in *rne* were also enriched in a number of Kyoto Encyclopedia of Genes and Genomes (KEGG) pathways, including ribosome, DNA replication, homologous recombination, mismatch repair, and base excision repair (Fig. 4c).

Among the genes downregulated in *rne* versus wild-type plants, the most highly enriched GO terms were photosynthesis (biological process), thylakoid (cellular component), and tetrapyrrole binding (molecular function) (Fig. 4b). The top six KEGG pathways enriched in downregulated genes were photosynthesis, starch and sucrose metabolism, phorosynthesis-antenna proteins, glyoxylate and dicarboxylate metabolism, plant-pathogen interaction, and carbon fixation in photosynthetic organisms (Fig. 4d). These results suggest that the expression of photosynthesis-associated nuclear genes (*PhANG*s) is repressed in the *rne* mutant and that *PhANG*s with reduced expression in *rne* primarily functioned in PSI and PSII, light harvesting, carbon fixation, and electron carrier processes (Table S4).

Discussion

Leaf color mutants are valuable genetic resources for Chinese cabbage breeding

As a group, the *Brassicas* are one of the most important vegetables worldwide and include Chinese cabbage, pakchoi (*Brassica rapa* L. ssp. *chinensis*), cabbage (*Brassica oleracea* var. *capitata*), cauliflower (*Brassica oleracea* var. *botrytis*), and others. Leaf color is one of the most important agronomic traits for *Brassica* vegetables; it is closely associated with consumer choice and affects the

RETRACTED ARTICLE

vegetables' quality and marketability. Although leaf color development is well studied in Arabidopsis, maize and rice, the essential genes and regulatory mechanisms that determine leaf color in *Brassica* vegetables remain elusive.

There has been recent progress in understanding the color development of *Brassica* vegetables. The stay-green genes *Brnye1* and *Brnym1* have been identified and analyzed in pakchoi and Chinese cabbage, respectively (Wang et al. 2018, 2020). The *Or* gene is known to cause weakly pigmented or unpigmented tissues to turn orange in cauliflower (Li et al. 2001), and the *Br-Or* gene was mapped and shown to confer orange inner leaf color in Chinese cabbage (Feng et al. 2012). Molecular markers derived from *Br-or* and *BrCRISTO1* cause orange-colored inner leaves in *Brassica rapa* (Zhang et al. 2013; Lee et al. 2014; Zou et al. 2016), and loss of *BrCRISTO1* function confers orange color to the inner leaves of Chinese cabbage (Su et al. 2015; Zhang et al. 2015). In addition, *BrPur* and *BrMYB2* have been mapped and shown to be tightly linked to the purple-leaf phenotype (Wang et al. 2014; He et al. 2020). Nevertheless, other genes that regulate leaf color remain to be discovered in Chinese cabbage. Although a pale-green EMS mutant was shown to harbor a mutation in the plastid gene *Rps4* (Tang et al. 2018), no nuclear genes that control pale-green traits have been reported previously in *Brassica*. In this study, an EMS Chinese cabbage mutant with pale-green leaves was used to map the gene *BrRNE*, which is essential for leaf color development (Fig. 1a–d, Fig. 2a). In addition, the regulatory mechanism by which BrRNE influences chloroplast development in Chinese cabbage was analyzed.

Plastid RNA processing is controlled by the nuclear gene *RNE* in *Brassica*

Chloroplasts evolved from cyanobacterial ancestors by endosymbiosis. They have their own genome and gene expression system including bacterial-type transcription and translation machineries (Harris et al. 1994; Dyall et al. 2004) that perform intron splicing (Zhang et al. 2017a, b), post-transcriptional cleavage of polycistronic mRNA into monocistronic units (RNA cutting) (Westhoff and Herrmann 1988), tRNA modification in translation (Liu et al. 2020) and ribosomal RNA processing (Liu et al. 2015). The steps of each process are coordinated, and defects in these processes affect chloroplast development, leading to distinct albino or pale-green phenotypes. RNase E/G type endoribonucleases have an essential role during plastid RNA cleavage in higher plants (Stoppel and Meurer 2012).

RNE is an endoribonuclease that has been studied primarily in *Escherichia coli*, where it plays a prominent role in the processing and degradation of RNA (Schein et al. 2008). An RNase E-like protein in higher plants was first characterized in Arabidopsis (Schein et al. 2008; Mudd et al. 2008). Arabidopsis RNE is present in the chloroplast, cleaves RNA similarly to the *E. coli* enzyme, and is essential for chloroplast development and autotrophic growth (Schein et al. 2008; Mudd et al. 2008). RNE participates in the intercistronic processing of primary transcripts from chloroplast operons, and *rne* plants show plastid ribosome deficiency because of the disturbed maturation of a transcript that encodes essential ribosomal proteins. Nonetheless, RNE is not essential for the survival of Arabidopsis plants growing in soil (Walter et al. 2010). Although RNE lacks a degradosome homolog in plant plastids compared with that in *E. coli* (Schein et al. 2008; Mudd et al. 2008; Stoppel et al. 2012), RHON1 directly interacts with RNE in the same high-molecular-weight (HMW) multiprotein complex, binding to single-stranded (ss) RNA to ensure the efficient processing of plastid transcripts by RNE in Arabidopsis (Stoppel et al. 2012). At present, little is known about RNE in other crops. In our study, *rne* plants grew autotrophically and survived well in soil and MS medium containing sucrose, but they could not survive in MS medium without sucrose under autotrophic conditions (Fig. 1a–b, Figure S2), This result suggests that the survival of *rne* plants may depends on carbon source supplementation. BrRNE was localized to the chloroplast (Fig. 3a) and cleaved chloroplast operons into monocistronic units (Fig. 3c). Its mutation reduced plastid protein translation levels (Fig. 3b), affecting chloroplast development and resulting in pale-green leaves in Chinese cabbage (Fig. 1a–d, l–n).

Aberrant plastid RNA cleavage causes strong retrograde signaling

It is now widely accepted that chloroplasts evolved from free-living prokaryotic organisms that were capable of photosynthesis (McFadden 2001). After serial endosymbiotic events, most genes encoding chloroplast proteins reside in the nucleus (Jarvis 2001). Chloroplast development is regulated by the coordinated expression of both chloroplast and nuclear genes (Pogson and Albrecht 2011). Signaling between the chloroplasts and the nucleus is bidirectional (Jung and Chory 2010). In anterograde regulation, the biogenesis and homeostasis of chloroplasts are controlled by their own genetic system in coordination with the nucleocytosolic system (Jarvis and López-Juez 2013). In retrograde signaling, nuclear gene expression is regulated as a result of signals generated in the plastids (Jung and Chory 2010). In the absence of chloroplast development, the expression of nuclear genes that encode chloroplast proteins such as light harvesting complex proteins is repressed (Jung and Chory

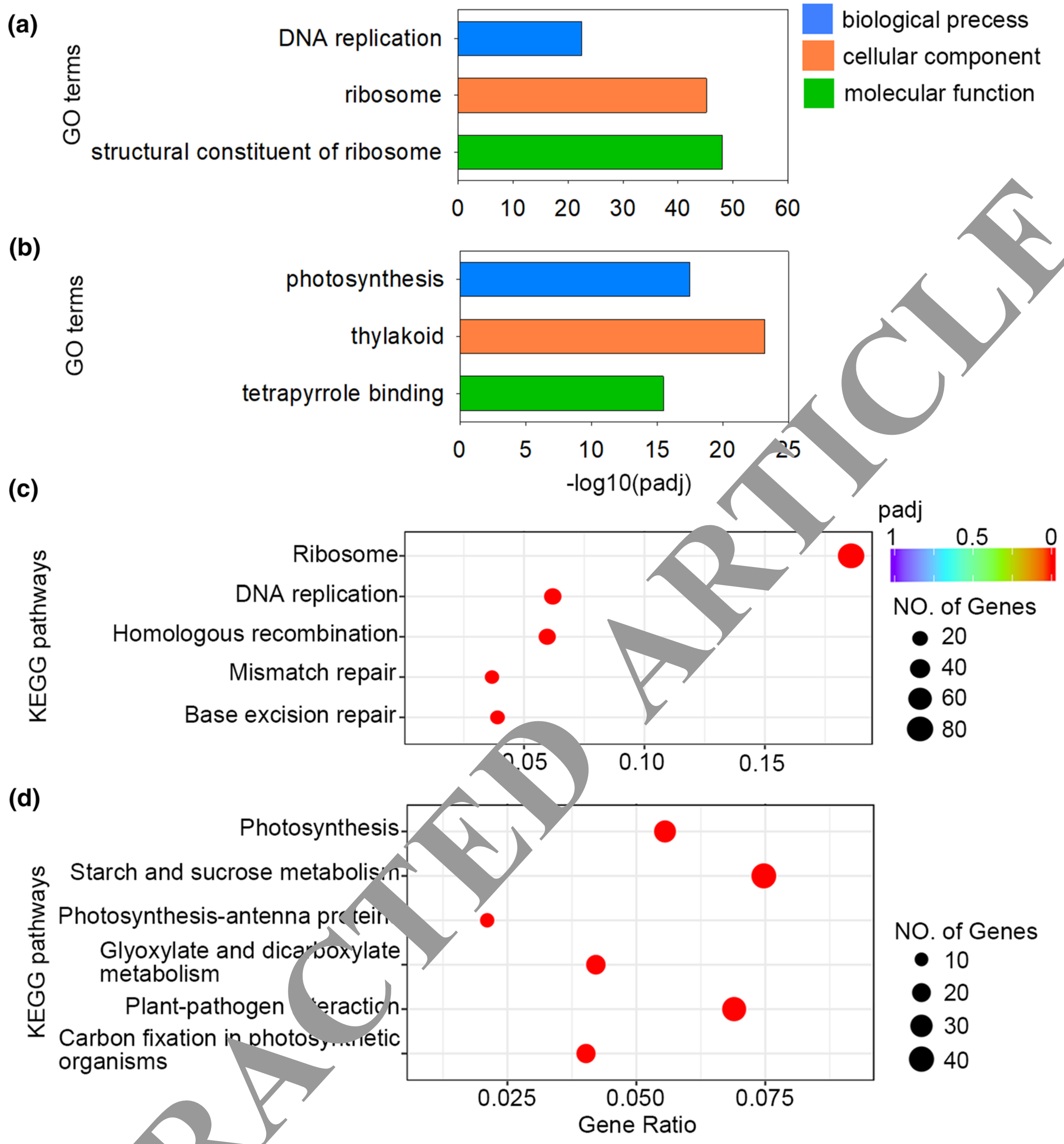

Fig. 4 RNA-seq ana[illegible]ysis of n[illegible]us-encoded genes in wild-type and *rne* plants at the [illegible] stage. **a**–**b** Gene ontology analysis of genes with increased ([illegible]) an[illegible]duced (**b**) expression levels in *rne* compared with the wild-type. **c**–**d** KEGG pathway analysis of genes with increased (**c**) and reduced (**d**) expression levels in *rne* compared with the wild-type

RETRACTED ARTICLE

2010; Liu [illegible]l. 20[illegible]0). To date, retrograde signaling caused by i[illegible]ired p[illegible]ocessing of plastid operons has not been r[illegible]orte[illegible] The size and organization of the *B. rapa* plastid gen[illegible] (Figure S6) were similar to those of the chloroplast chrom[illegible]somes of most land plants (Palmer 1985; Shinozaki et al. 1986). Our RNA-seq data showed that a large number of nuclear genes in the ribosome and DNA replication pathways were significantly upregulated in the *rne* mutant (Fig. 4c), whereas the expression of nuclear genes involved in carbon metabolism and photosynthetic pathways was markedly decreased (Fig. 4d). These results indicate that RNA cleavage triggers chloroplast-to-nucleus retrograde signaling.

In this study, we demonstrated that a functional defect of *BrRNE* leads to impaired RNA processing, thereby affecting the translation of plastid genes in the chloroplasts (Fig. 3b–c). RNA-seq analysis revealed that the expression of nuclear genes was dramatically altered in the *rne* mutant, with increased expression levels of genes involved in ribosome composition, DNA replication, and mismatch repair pathways. This result suggests suggesting that the nucleus may compensate for the impaired translation of

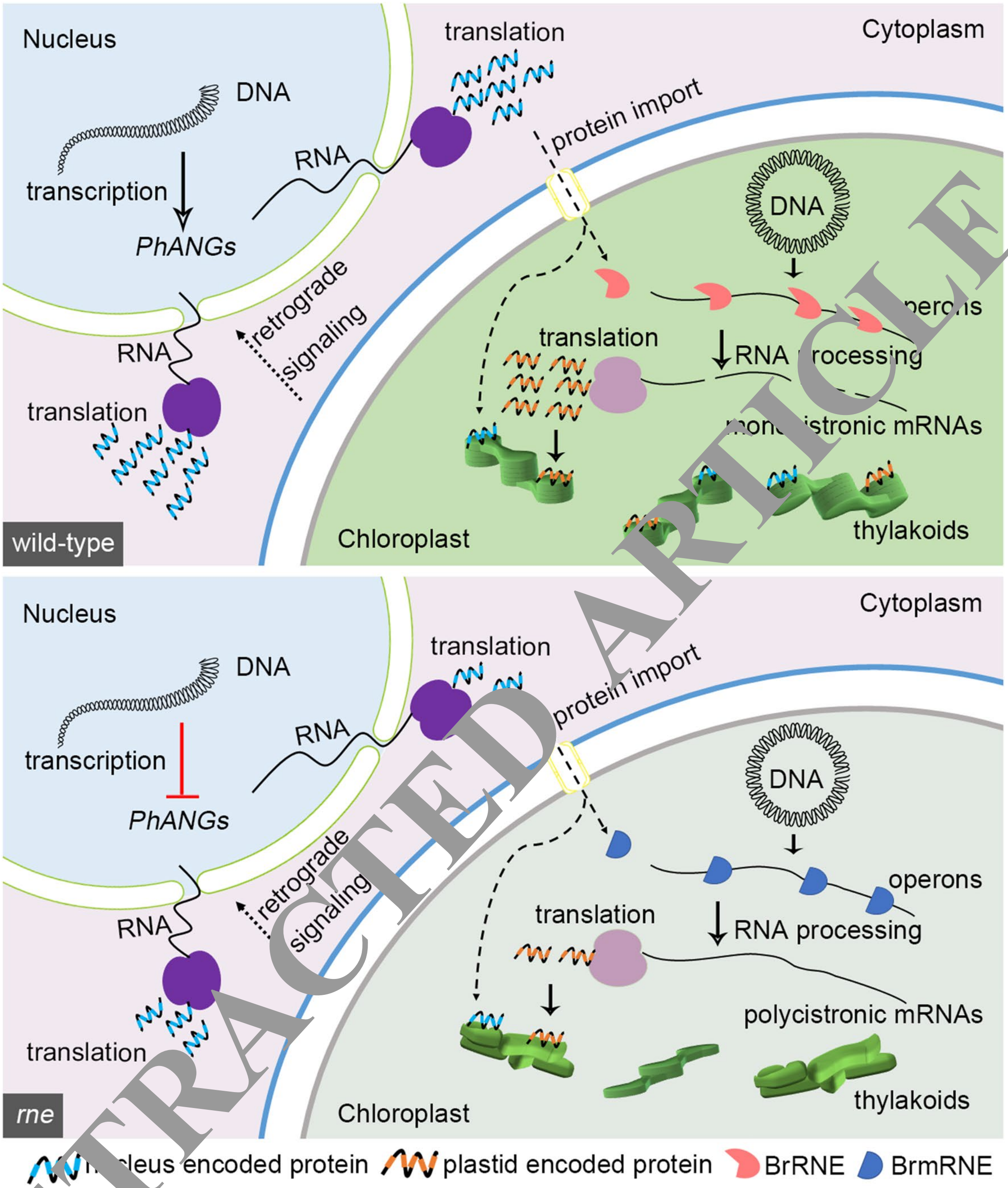

RETRACTED ARTICLE

Fig. 5 A proposed working model of the molecular function of RNE. In the wild-type, BrRNE cleaves polycistronic precursor transcripts into mature monocistronic mRNAs in chloroplasts. In *rne*, the defective function of BrRNE results in impaired RNA processing. Thus, aberrant chloroplast RNA cleavage leads to an abnormal thylakoid system and a pale-green phenotype. In addition, responding to retrograde signaling, the transcript levels of photosynthesis-associated nuclear genes (*PhANG*s) in the nucleus are reduced in *rne*

plastid genes and restore plastid RNA cleavage in the *rne* mutant (Fig. 3c). On the other hand, as reported by Liu et al. (2020), the suppression of *PhANG*s in *rne* may facilitate plant survival by conserving energy through reduced plant growth (Fig. 1a–d, Figure S1).

Taken together, our results suggest the following working model of the regulatory mechanism that underlies BrRNE function in wild-type and *rne* plants (Fig. 5). In

RETRACTED ARTICLE

the wild-type, BrRNE cleaves polycistronic precursor transcripts into mature monocistronic mRNAs in chloroplasts. In *rne*, the defective function of BrRNE results in impaired RNA processing. Aberrant chloroplast RNA cleavage then leads to reduced levels of photosynthetic proteins, resulting in an abnormal thylakoid system and a pale-green phenotype. In addition, responding to retrograde signaling, the transcript levels of *PhANG*s in the nucleus are reduced in *rne*, leading to smaller plants.

Supplementary Information The online version contains supplementary material available at https://doi.org/10.1007/s00122-021-03905-z.

Acknowledgements We appreciate the linguistic assistance provided by A&L Scientific Editing (www.alpublish.com) during the preparation of this manuscript. This work was financially supported in part by the National Natural Science Foundation of China (Grant No. 31801857, 31930098), the Natural Science Foundation of Hebei (C2019204201), the China Postdoctoral Science Foundation (2018M641668), the International Cooperation Project in the Science and Technology Support Program of Hebei (Grant No. 2019YX023A), the Innovative Research Group Project of Hebei Natural Science Foundation (C2020204111), the Program for Young Talents of Hebei Education Department (BJ2021024), and the Science and Technology Research Project of Universities in Hebei Province (BJ2019020).

Author contribution statement X.Z., W.M. and J.Z. conceived and designed the experiments. X.Z. and X.L. performed the experiments, X.Z. and W.M. wrote the manuscript, and X.L., Y.L. and D.F. surveyed the morphological characteristics. M.L., S.Z. and Y.L. performed the genetic analysis. S.S. reviewed the manuscript.

Declarations

Conflicts of interest The authors have no conflict of interest to declare.

Open Access This article is licensed under a Creative Commons Attribution 4.0 International License, which permits use, sharing, adaptation, distribution and reproduction in any medium or format, as long as you give appropriate credit to the original author(s) and the source, provide a link to the Creative Commons licence, and indicate if changes were made. The images or other third party material in this article are included in the article's Creative Commons licence, unless indicated otherwise in a credit line to the material. If material is not included in the article's Creative Commons licence and your intended use is not permitted by statutory regulation or exceeds the permitted use, you will need to obtain permission directly from the copyright holder. To view a copy of this licence, visit http://creativecommons.org/licenses/by/4.0/.

References

Abe A, Kosugi S, Yoshida K, Natsume S, Takagi H, Kanzaki H, Matsumura H et al (2012) Genome sequencing reveals agronomically important loci in rice using MutMap. Nat Biotechnol 30(2):174–178

Babiychuk E, Müller F, Eubel H, Braun HP, Frentzen M, Kushnir S (2003) Arabidopsis phosphatidylglycerophosphate synthase 1 is essential for chloroplast differentiation, but is dispensable for mitochondrial function. Plant J 33(5):899–909

Beale SI (2005) Green genes gleaned. Trends Plant Sci 10(7):309–312

Benjamini Y, Hochberg Y (1995) Controlling the false discovery rate: a practical and powerful approach to multiple testing. J R Stat Soc B 57:289–300

Chi W, Feng P, Ma J, Zhang L (2015) Metabolites and chloroplast retrograde signaling. Curr Opin Plant Biol 25:32–38

Chiu LW, Zhou X, Burke S, Wu X, Prior RL, Li L (2010) The purple cauliflower arises from activation of a MYB transcription factor. Plant Physiol 154(3):1470–1480

Dyall SD, Brown MT, Johnson PJ (2004) Ancient invasions: from endo-symbionts to organelles. Sci 304:253–257

Feng H, Li Y, Liu Z, Liu J (2012) Mapping of *or*, a gene conferring orange color on the inner leaf of the Chinese cabbage (*Brassica rapa* L. ssp. *pekinensis*). Mol Breeding [illegible]:235–244

Frick G, Su Q, Apel K, Armstrong GA (2003) An Arabidopsis *porB porC* double mutant lacking light-dependent NADPH: protochlorophyllide oxidoreductases B and C is highly chlorophyll-deficient and developmentally arrested. Plant J 35(2):141–153

Greiner S, Lehwark P, Bock R (2019) OrganellarGenomeDRAW (OGDRAW) version 1.3.1: expanded toolkit for the graphical visualization of organellar genomes. Nucleic Acids Res 47:W59–W64

Guindon S, Dufayard JF, Lefort V, Anisimova M, Hordijk W, Gascuel O (2010) New algorithms and methods to estimate maximum-likelihood phylogenies: assessing the performance of PhyML 3.0. Syst Biol 59(3):307–21

He Q, [illegible] Y, Zhao W, Li R, Zhang L (2020) The novel gene *BrMYB2*, located on chromosome A07, with a short intron 1 controls the purple-head trait of Chinese cabbage (*Brassica rapa* L.). Hortic Res 7:97

Harris EH, Boynton JE, Gillham NW (1994) Chloroplast ribosomes and protein synthesis. Microbiol Rev 58:700–754

Jarvis P (2001) Intracellular signalling: the chloroplast talks! Curr Biol 11(8):R307–R310

Jarvis P, López-Juez E (2013) Biogenesis and homeostasis of chloroplasts and other plastids. Nat Rev Mol Cell Biol 14(12):787–802

Jung HS, Chory J (2010) Signaling between chloroplasts and the nucleus: can a systems biology approach bring clarity to a complex and highly regulated pathway? Plant Physiol 152(2):453–459

Jung KH, Hur J, Ryu CH, Choi Y, Chung YY, Miyao A, Hirochika H, An G (2003) Characterization of a rice chlorophyll-deficient mutant using the T-DNA gene-trap system. Plant Cell Physiol 44(5):463–472

Lee S, Lee SC, Byun DH, Lee DY, Park JY, Lee JH, Lee HO, Sung SH, Yang TJ (2014) Association of molecular markers derived from the *BrCRTISO1* gene with prolycopene-enriched orange-colored leaves in *Brassica rapa*. Theor Appl Genet 127(1):179–191

Li L, Paolillo DJ, Parthasarathy MV, Dimuzio EM, Garvin DF (2001) A novel gene mutation that confers abnormal patterns of beta-carotene accumulation in cauliflower (Brassica oleracea var botrytis). Plant J. 26(1):59–67

Liu J, Zhou W, Liu G, Yang C, Sun Y, Wu W, Cao S et al (2015) The conserved endoribonuclease YbeY is required for chloroplast ribosomal RNA processing in Arabidopsis. Plant Physiol 168(1):205–221

Liu H, Ren D, Jiang L, Li X, Yao Y, Mi L, Chen W et al (2020) A natural variation in PLEIOTROPIC DEVELOPMENTAL DEFECTS uncovers a crucial role for chloroplast tRNA modification in translation and plant development. Plant Cell 32(7):2345–2366

Lu Y, Dai S, Gu A, Liu M, Wang Y, Luo S, Zhao Y et al (2016) Microspore induced doubled haploids production from Ethyl Methanesulfonate (EMS) soaked flower buds is an efficient strategy for mutagenesis in Chinese cabbage. Front Plant Sci 7:1780

Manjaya J (2009) Genetic improvement of soybean variety VLS-2 through induced mutations. Small 38:106–109

RETRACTED ARTICLE

Mao XZ, Cai T, Olyarchuk JG, Wei LP (2005) Automated genome annotation and pathway identification using the KEGG Orthology (KO) as a controlled vocabulary. Bioinformatics 21:3787–3793

McFadden GI (2001) Chloroplast Origin and Integration. Plant Physiol 125(1):50–53

Mudd EA, Sullivan S, Gisby MF, Mironov A, Kwon CS, Chung WI, Day A (2008) A 125 kDa RNase E/G-like protein is present in plastids and is essential for chloroplast development and autotrophic growth in Arabidopsis. J Exp Bot 59(10):2597–2610

Muramoto T, Kohchi T, Yokota A, Hwang I, Goodman HM (1999) The Arabidopsis photomorphogenic mutant *hy1* is deficient in phytochrome chromophore biosynthesis as a result of a mutation in a plastid heme oxygenase. Plant Cell 11(3):335–347

Nagata N, Tanaka R, Satoh S, Tanaka A (2005) Identification of a vinyl reductase gene for chlorophyll synthesis in *Arabidopsis thaliana* and implications for the evolution of *Prochlorococcus* species. Plant Cell 17(1):233–240

Palmer JD (1985) Comparative organization of chloroplast genomes. Annu Rev Genet 1:325–354

Parks BM, Quail PH (1991) Phytochrome-deficient *hy1* and *hy2* long hypocotyl mutants of Arabidopsis are defective in phytochrome chromophore biosynthesis. Plant Cell 3(11):1177–1186

Paul J, Enrique LJ (2014) Biogenesis and homeostasis of chloroplasts and other plastids. Nat Rev Mol Cell Bio 15:147–147

Pogson BJ, Albrecht V (2011) Genetic dissection of chloroplast biogenesis and development: an overview. Plant Physiol 155:1545–1551

Schein A, Sheffy-Levin S, Glaser F, Schuster G (2008) The RNase E/G-type endoribonuclease of higher plants is located in the chloroplast and cleaves RNA similarly to the E. coli enzyme. RNA. 14(6):1057–1068

Shinozaki K, Ohme M, Tanaka M, Wakasugi T, Hayashida N, Matsubayashi T, Zaita N et al (1986) The complete nucleoti[illegible] sequence of the tobacco chloroplast genome: its gene organization and expression. EMBO J 5(9):2043–2049

Singh R, Ikehashi H (1981) Monogenic male-sterility in ri[illegible] induction, identification and inheritance. Crop Sci 21:[illegible]86–289

Stoppel R, Meurer J (2012) The cutting crew - [illegible] nucleases are key players in the control of plastid gene expressi[illegible] J Exp Bot 63(4):1663–1673

Su T, Yu S, Zhang JWF, Yu Y, Zhang D, Zha[illegible] X, Wang W (2015) Loss of function of the carotenoid isomeras[illegible] gene *BrCRTISO* confers orange color to the inner leaves of Chi[illegible] cabbage (*Brassica rapa* L. ssp. *pekinensis*). Plant [illegible] Biol Rep 33:648–659

Sundberg E, Slagter JG, Fridborg I, [illegible], Robinson C, Coupland G (1997) *ALBINO3*, an Arab[illegible]dopsis nuclear gene essential for chloroplast differen[illegible]n, encodes a chloroplast protein that shows homology to [illegible]teins [illegible]t in bacterial membranes and yeast mitochondri[illegible] Pla[illegible] Cell 9(5):717–730

Tanaka A, Tanaka [illegible] (2006) Ch[illegible]ophyll metabolism. Curr Opin Plant Biol 9(3):24[illegible]

Tanaka A, It[illegible] H, Tana[illegible] R, Tanaka NK, Yoshida K, Okada K (1998) Chlorophyll [illegible] oxygenase (CAO) is involved in chlorophyl[illegible] [illegible]ation from chlorophyll a. Proc Natl Acad Sci USA 95(21):1[illegible]19–1[illegible]2723

Tang [illegible] Wang [illegible] Zhang Y, Huang S, Liu Z, Fei D, Feng H (2018) A mis[illegible] mutation of plastid RPS4 is associated with chlorophyll [illegible]ficiency in Chinese cabbage (Brassica campestris ssp. pekinensis[illegible] BMC Plant Biol. 18(1):130

Terry MJ, Kendrick RE (1999) Feedback inhibition of chlorophyll synthesis in the phytochrome chromophore-deficient *aurea* and *yellow-green-2* Mutants of Tomato. Plant Physiol 119(1):143–152

Walter M, Piepenburg K, Schöttler MA, Petersen K, Kahlau S, Tiller N, Drechsel O, Weingartner M, Kudla J, Bock R (2010) Knockout of the plastid RNase E leads to defective RNA processing and chloroplast ribosome deficiency. Plant J 64(5):851–863

Wang W, Zhang D, Yu S, Liu J, Wang D, Zhang F, Yu Y, Zhao X, Lu G, Su T (2014) Mapping the *Br Pu*r gene for purple leaf color on linkage group A03 of *Brassica rap*a. Euphytica 199:293–302

Wang N, Liu Z, Zhang Y, Li C, Feng H (2018) Identification and fine mapping of a stay-green gene (Brnye1) in [illegible]akchoi (Brassica campestris L. ssp. chinensis). Theor. Appl. G[illegible] 1[illegible](3):[illegible]73–684

Wang N, Zhang Y, Huang S, Liu Z, Li C, Feng H ([illegible]20) [illegible]efect in Brnym1, a magnesium-dechelatase protein, causes [illegible] stay-green phenotype in an EMS-mutagenized [illegible]inese ca[illegible]bage (*Brassica campestris* L. ssp. *pekinensis*) lin[illegible] Hort[illegible] Res [illegible]:8

Westhoff P, Herrmann RG (1988) [illegible]omplex RN[illegible] maturation in chloroplasts. Eur J Biochem 171:[illegible]51–564

Young MD, Wakefield MJ, Sm[illegible]yth [illegible] Oshla[illegible]ck A (2010) Gene ontology analysis for RNA-se[illegible]ccou[illegible]g for selection bias. Genome Biol 11:R14

Zhang J, Li H, Zhang [illegible] Hui M, W[illegible]g Q, Li L, Zhang L (2013) Fine mapping and i[illegible]enti[illegible]tion of candidate *Br-or* gene controlling orange head [illegible] Chinese [illegible]bbage (*Brassica rapa* L. ssp. *pekinensis*). Mol Bree[illegible]ng 32:799–805

Zhang J, Yua[illegible] H, [illegible]ogson BJ, Zhang L, Li L (2015) Molecular characte[illegible]ation and transcriptome analysis of orange head C[illegible]inese cabb[illegible]ge (*Brassica rapa* L. ssp. *pekinensis*). Planta 24[illegible]394

Zhang J, [illegible]iao J, Li Y, Su B, Xu H, Shan X, Song C, Xie J, Li R (2017a) PDM[illegible] a pentatricopeptide repeat-containing protein, affects chloroplast development. J Exp Bot 68(20):5615–5627

[illegible]ng L, Su W, Tao R, Zhang W, Chen J, Wu P, Yan C et al (2017b) RNA sequencing provides insights into the evolution of lettuce and the regulation of flavonoid biosynthesis. Nat Commun 8(1):2264

Zhao Y, Wang M, Zhang YZ, Du LF, Pan T (2001) Chloroplast composition and structure differences in a chlorophyll-reduced mutant of oilseed rape seedlings. Acta Bot Sin 43(8):877–880

Zhao W, Yang X, Yu H, Jiang W, Sun N, Liu X, Zhang X, Wang Y, Gu X (2014) RNASeq-based transcriptome profiling of early nitrogen deficiency response in cucumber seedlings provides new insight into the putative nitrogen regulatory network. Plant Cell Physiol 56:455–467

Zhao C, Wang Y, Chan KX, Marchant DB, Franks PJ, Randall D, Tee EE et al (2019) Evolution of chloroplast retrograde signaling facilitates green plant adaptation to land. Proc Natl Acad Sci USA 116(11):5015–5020

Zou CL, Zheng Y, Wang P, Zhang X, Wang YH, Liu ZY, Feng H (2016) Fine mapping and characterization of the *or* gene in Chinese cabbage (*Brassica rapa* L. ssp *pekinensis*). Genet Mol Res 15(2)

Publisher's Note Springer Nature remains neutral with regard to jurisdictional claims in published maps and institutional affiliations.
